# Evaluation of a self-management programme for congestive heart failure patients: design of a randomised controlled trial

**DOI:** 10.1186/1472-6963-6-91

**Published:** 2006-07-20

**Authors:** Esther STF Smeulders, Jolanda CM van Haastregt, Elisabeth FM van Hoef, Jacques ThM van Eijk, Gertrudis IJM Kempen

**Affiliations:** 1 Maastricht University, Faculty of Health Sciences, Department of Health Care Studies, P.O. Box 616, 6200 MD Maastricht, The Netherlands

## Abstract

**Background:**

Congestive heart failure (CHF) has a substantial impact on care utilisation and quality of life. It is crucial for patients to cope with CHF adequately, if they are to live an acceptable life. Self-management may play an important role in this regard. Previous studies have shown the effectiveness of the 'Chronic Disease Self-Management Program' (CDSMP), a group-based cognitive behavioural programme for patients with various chronic conditions. However, the programme's effectiveness has not yet been studied specifically among CHF patients. This paper presents the design of a randomised controlled trial to evaluate the effects of the CDSMP on psychosocial attributes, health behaviour, quality of life, and health care utilisation of CHF patients.

**Methods/Design:**

The programme is being evaluated in a two-group randomised controlled trial. Patients were eligible if they had been diagnosed with CHF and experienced slight to marked limitation of physical activity. They were selected from the Heart Failure and/or Cardiology Outpatient Clinics of six hospitals. Eligible patients underwent a baseline assessment and were subsequently allocated to the intervention or control group. Patients allocated to the intervention group were invited to attend the self-management programme consisting of six weekly sessions, led by a CHF nurse specialist and a CHF patient. Those allocated to the control group received care as usual. Follow-up measurements are being carried out immediately after the intervention period, and six and twelve months after the start of the intervention.

An effect evaluation and a process evaluation are being conducted. The primary outcomes of the effect evaluation are self-efficacy expectancies, perceived control, and cognitive symptom management. The secondary outcome measures are smoking and drinking behaviour, Body Mass Index (BMI), physical activity level, self-care behaviour, health-related quality of life, perceived autonomy, symptoms of anxiety and depression, and health care utilisation. The programme's feasibility is assessed by measuring compliance with the protocol, patients' attendance and adherence, and the opinions about the programme.

**Discussion:**

A total number of 318 patients were included in the trial. At present, follow-up data are being collected. The results of the trial become clear after completion of the data collection in January 2007.

**Trial Registration:**

Trialregister () ISRCTN88363287.

## Background

Effective management of congestive heart failure (CHF) is one of the major challenges in health care today. The number of CHF patients is substantial: CHF affects up to 20% of the rapidly increasing population of persons aged 65 years and over. Of every three individuals aged 55 years and over, one will develop CHF during their lifetime, with only 35% surviving 5 years following diagnosis [1]. In the aging population of the Western world, this implies a further expansion of the CHF epidemic in the near future [2,3]. CHF has a substantial impact on care utilisation, daily functioning, and quality of life [4,5].

There is increasing evidence that self-management programmes for CHF patients have positive effects on self-care behaviour and quality of life [6-9]. Managing CHF is a complex task, and comparable to diabetes in that it touches nearly every important aspect of daily life [10]. It is therefore crucial for patients to cope with CHF adequately, if they are to live an acceptable life. Exercise intolerance, often experienced by CHF patients, may lead to activity restriction and progressive functional deterioration. This downward spiral may be interrupted by physical activity, which may improve functional capacity and quality of life [11]. In addition, diet and lifestyle changes, such as limiting salt intake and balancing rest and activity, are important to prevent complications and hospitalisation, and to slow the disease progression [12,13]. Self-management skills such as fatigue management, breathing techniques, and relaxation can play an important role in everyday CHF management [14].

To teach patients how to adequately self-manage the consequences of their chronic disease for daily life, Lorig and colleagues developed the "Chronic Disease Self-Management Program" (CDSMP) at Stanford University in California (USA) [14-18]. The CDSMP is a structured group-based self-management programme for people with chronic conditions, addressing general management problems for various chronic conditions. The CDSMP is based on the self-efficacy theory developed by Bandura [19] and incorporates skills mastery, reinterpretation of symptoms, modelling, and social persuasion to enhance self-efficacy expectancies [16,18]. Self-efficacy expectancies refer to the belief that one can successfully perform intended behaviours [19], and are powerful determinants of behavioural change [20]. There are indications that low levels of self-efficacy expectancies predict functional decline in CHF patients [21]. Improving self-efficacy expectancies may therefore change health behaviour, improve health-related quality of life, and reduce care utilisation by CHF patients.

Previous studies have found favourable effects of the CDSMP (e.g. on self-efficacy expectancies and hospitalisation) in arthritis patients [22] and in groups of patients with various chronic conditions attending the same class [17,23], even after one and two years [15,16]. However, the effects of the programme have not yet been studied specifically among CHF patients. Our basic assumption is that increasing levels of self-efficacy expectancies will help CHF patients to interrupt the downward spiral of functional disability and demoralisation after their cardiac event. Therefore, we decided to evaluate the effects of the CDSMP on psychosocial attributes (e.g. self-efficacy expectancies), health behaviour, quality of life, and health care utilisation of CHF patients. The current paper presents the design of a multi-centre randomised controlled trial evaluating the CDSMP among CHF outpatients in the south of the Netherlands. The study involves an effect evaluation and an evaluation of the intervention process. The process analysis is expected to yield information on factors increasing or hampering the effectiveness and implementation of the programme in outpatient care for CHF patients, which may be crucial later on if the group programme turns out to be effective.

### Aims

The main objectives of the current study are: (1) to assess the effects of the cognitive behavioural CDSMP intervention on psychosocial attributes, health behaviour, quality of life, and health care utilisation of CHF patients, and (2) to assess the feasibility of the CDSMP intervention according to patients and leaders of the programme.

## Methods/Design

### Design

**Figure 1:**
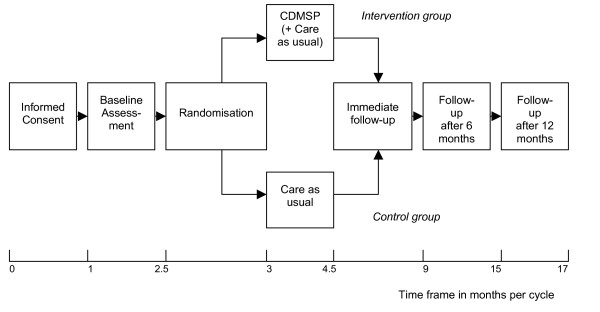
Study design.

This evaluation study is designed as a two-group randomised controlled trial in which patients are randomly allocated to the intervention or control group (the latter receiving usual care). Data are collected at baseline before the start of the intervention, immediately after the programme (six weeks after the start), and six and twelve months after the start of the programme (see figure 1). Data are gathered by means of self-administered questionnaires and telephone interviews. Various measures are taken to ensure blinding in the data collection process. Questionnaires are collected anonymously and sorted by number. The telephone interviews are conducted independently by trained interviewers, who are unaware to which group patients have been allocated.

For practical reasons, the study consists of eleven consecutive cycles, with one or more CDSMP classes in each cycle. In each cycle, patients are included and randomly allocated to the intervention group or control group. The first cycle started in July 2004 and the final one in September 2005. A total of 21 CDSMP classes (with a parallel control group) were conducted in the period October 2004 – January 2006, in six hospitals. At least two CDSMP classes were conducted in each participating hospital (range 2–6). The Medical Ethics Committee of Maastricht University/University Hospital Maastricht granted approval to conduct this trial. Furthermore, local feasibility of the study design was assessed by each of the local Medical Ethics Committees in the other five participating hospitals.

### Participants

Patients were selected by cardiologists or CHF nurse specialists of the Heart Failure and/or Cardiology Outpatient Clinics in the six participating hospitals. Patients were eligible for the study if they met the following four criteria: (1) having been diagnosed with CHF based on a systolic dysfunction (left ventricular ejection fraction (LVEF) < 40% with New York Heart Association (NYHA) Class II-III symptoms; slight to marked limitation of physical activity) or a diastolic dysfunction (LVEF ≥ 40% with NYHA Class II-III symptoms plus an additional hospital admission based on decompensatio cordis after CHF diagnosis); (2) having been a CHF patient for at least six months at the start of the intervention period, (3) being able to understand, write, and speak Dutch, and (4) being willing to give informed consent to participate in the study. Patients were excluded if they were participating in other scientific research. All eligible patients received written information about the programme and the study. Patients who signed the informed consent form were invited to participate in the baseline measurement.

### Randomisation

In each cycle, patients were randomised after completion of the baseline measurement if a sufficiently large group of eligible patients per hospital could be formed. We decided that at least eight patients per group had to be allocated to the intervention group, because CDSMP classes normally consist of at least eight participants, for reasons of group dynamics [16]. Eligible patients therefore had a slightly higher chance to be allocated to the intervention group (59%) than to the control group (41%) during the total recruitment period. The maximum number of patients allocated to each CDSMP class was set at 14. The randomisation procedure was performed by an independent researcher by random computerised allocation using SPSS 12.0 for Windows.

### Intervention

The CDSMP consists of six weekly sessions (two and a half hours per session) and emphasises patients' central role and responsibility in managing their illness [16]. The programme aims to teach patients three types of self-management techniques: dealing with the medical management of the disease (medical management); maintaining, changing, and creating new meaningful behaviours or life roles (role management), and dealing with the emotional consequences of having a chronic condition (emotional management) [18]. These self-management techniques are taught by means of skills mastery through weekly action planning and feedback on progress, modelling of self-management behaviours and problem-solving strategies, reinterpretation of physiological symptoms, and social persuasion through group support and guidance for individual self-management efforts [16,18]. Table 1 shows the CDSMP activities per session. While developing the CDSMP, Lorig and colleagues published the book *Living a healthy life with chronic conditions *[14], as a reference source for the material covered in the sessions. The patients received this book at the first session of the programme. Before the start of the study, the original programme had been translated into Dutch, including *Living a healthy life with chronic conditions *[14]. Some minor adjustments were made in this translated version of the reference book, based on differences between the American and Dutch settings (e.g. with regard to advance directives).

In the original version of the programme, CDSMP classes are led by two trained lay leaders. For the present study, however, we opted for a combination of a 'professional leader' (a CHF nurse specialist) and a 'peer leader' (CHF patient), acting as a role model for the other CHF patients in the class. The reason was that we expect a programme which is led by at least one professional leader to be easier to implement in regular health care than a programme led by two lay leaders only. Furthermore, CHF is a serious disease with unexpected exacerbations and intercurrent health problems. This means that peer leaders may only be able to conduct the programme for a limited period, which could form a threat to the continuity of the programme.

In four of the six hospitals, CHF nurse specialists and CHF patients were trained as leaders. These leaders also conducted CDSMP classes in the two other participating hospitals. A total of twelve professionals and eleven CHF patients received four days of training with the CDSMP protocol in the period April 2004 – November 2005. Four of the twelve professional leaders and two of the eleven peer leaders did not implement the programme in the intervention period, due to work, health, or private issues. Each of the groups in which the leaders were trained (n = 4 to 6) included both professional leaders and peer leaders, who practised together, to provide an optimal training situation. The leader training was provided by one of the researchers (author ES) and a CHF nurse specialist of the University Hospital Maastricht in the Netherlands. Both had been instructed as master trainers at Stanford University by Lorig and colleagues in March 2004. The nurse master trainer also conducted several classes in the intervention period.

### Measurements in the effect evaluation

#### Primary outcome variables

The primary outcomes of the effect evaluation are psychosocial attributes, conceptualised as self-efficacy expectancies, perceived control, and cognitive symptom management. The primary outcomes are assumed to be directly influenced by the self-management programme.

Self-efficacy expectancies were assessed by two scales; one is a generic scale, allowing us to compare our results with other studies, whereas the other is a specific scale for cardiac patients, used to improve the sensitivity to change in our study population. General self-efficacy expectancies were measured by the 'General Self-Efficacy Scale' (GSES) [20], which determines the extent to which people believe that they can successfully perform a particular behaviour. Cardiac self-efficacy expectancies were measured using the 'Cardiac Self-Efficacy Questionnaire' [24], which has also been used in other Dutch research among CHF patients [25,26].

Perceived control was assessed by means of the mastery scale developed by Pearlin and Schooler [27]. This scale measures "the extent to which one regards one's life chances as being under one's own control, in contrast to being fatalistically ruled" [27]. The psychometric properties of the Dutch versions of the GSES and the perceived control scale had proved satisfactory in previous studies [21,28,29].

Cognitive symptom management was assessed by the 'Coping with symptoms scale' developed by Lorig and colleagues [30], which measures how often respondents use cognitive symptom management techniques when they feel depressed or experience pain or other unpleasant symptoms. The original 6-item scale was slightly modified into a 5-item scale by omitting the first two items and adding one extra item about the use of breathing techniques as a way to deal with unpleasant symptoms (Elzen et al., evaluation of the CDSMP among chronically ill elderly people in the Netherlands, submitted). Table 2 provides an overview of the outcome variables of the effect evaluation.

#### Secondary outcome variables

The outcome measures considered to be indirectly influenced by the programme (secondary outcomes) are health behaviour, quality of life, and health care utilisation (see table 2).

Health behaviour was operationalised into three measurements: (1) smoking and drinking behaviour and Body Mass Index (BMI) (kg/m2), (2) physical activity level, and (3) self-care behaviour. Smoking and drinking behaviour were assessed by asking the patients whether they smoked or drank and in what amounts (i.e. mean number of cigarettes/cigars/pipes smoked per day and mean number of alcoholic drinks per week, respectively). Physical activity level was measured by a modified version of the 'Physical activities scale' developed by Lorig and colleagues [30]. This scale comprises questions about the frequency of walking for exercise, swimming, and bicycling in the preceding month. In addition, respondents were given an opportunity to name alternative exercises in an open-ended question, such as gardening, housekeeping, etc. Finally, CHF-specific self-care behaviour was measured with the 'European Heart Failure Self-Care Behaviour Scale' (EHFScBS), a scale developed in the Netherlands, with good psychometric properties [31].

Quality of life was conceptualised as health-related quality of life, perceived autonomy, and symptoms of anxiety and depression. Health-related quality of life was measured using both generic and disease-specific instruments; the 'RAND 36-item Health Survey' (RAND-36) [32] and the 'Kansas City Cardiomyopathy Questionnaire' (KCCQ) [33]. Perceived autonomy was assessed by one item using a visual analogue scale to measure to what extent the patients were able to arrange their lives the way they would like to (I do not feel I can do so at all – I strongly feel I can do so). Symptoms of anxiety and depression were measured by the 'Hospital Anxiety and Depression Scale' (HADS) [34,35], which is considered to be unbiased by coexisting general medical conditions [36]. The psychometric properties of the Dutch versions of the RAND-36 and the HADS have proved to be satisfactory in previous studies [36,37].

Finally, health care utilisation was measured by patients' self-reports on the number of contacts with a general practitioner, cardiologist, other medical specialist or CHF nurse specialist, the number of hospitalisation days, the reason for hospitalisation, and the number of visits to accident and emergency departments.

#### Covariates

Some additional variables were assessed to characterise the study population and interpret the outcomes of the trial (see table 2). Socio-demographics (gender, age, marital status, living arrangements, education level, and job situation) were assessed at baseline. In addition, two health-related variables were assessed at baseline: co-morbidity using a 19-item checklist of medical conditions [21,38] and cognitive status using the Dutch version of the Telephone Interview for Cognitive Status (TICS) [39,40].

### Measurements in the process evaluation

To determine the feasibility of the programme, four process outcomes were assessed by means of structured evaluation forms: (1) the extent to which the programme was performed according to protocol and the time spent on the sessions, (2) patients' attendance, (3) overall patient adherence and specific adherence with regard to homework assignments, and (4) the opinions of the patients and leaders about the programme. Table 3 presents the outcome variables of the process evaluation.

With regard to compliance with the protocol, the leaders recorded after each session whether they had exceeded specified time limits per activity, skipped, shortened or added parts to activities, or used different training techniques than specified in the protocol. In addition, they recorded preparation time, travel time, time spent on the session, and activities resulting from the session (e.g. answering patients' questions afterwards)

The leaders reported on patient attendance and adherence at group level. Absent patients were phoned by the professional leader to ask for the reason of their absence. Overall adherence (i.e. patients' efforts during the programme sessions) was also assessed at group level, using a 5-point scale (very good – very poor). After completing the programme, both leaders and patients reported on the adherence with regard to homework assignments (e.g. drawing up weekly action plans).

Immediately after the programme, patients who had attended at least three of the six sessions received a structured evaluation form to assess their opinion about the CDSMP. They were asked to what extent they had benefited from the programme (5-point scale; not benefited – benefited very much), and which parts they did or did not appreciate. In addition, patients were asked: (1) to give report marks for the quality of the professional leader and the peer leader, and the programme itself (10-point scale; 1 to 10), (2) whether they would recommend the CDSMP to other CHF patients or to patients with other chronic diseases (e.g. diabetes mellitus), and (3) whether the programme had helped them improve specific health behaviours and coping strategies, compared with the situation before they attended the programme. Finally, the patients were asked to suggest improvements to the CDSMP.

The leaders' opinions about the programme were assessed after each session (report mark on the session, strong and weak points of the session) and after they had completed the programme (report mark on their own skills as leader and questions about the perceived benefits to the patients). At the end of the intervention period (January 2006), all leaders were invited for a structured group interview. During this meeting, the leaders evaluated the feasibility of the protocol in more general terms and made recommendations to improve the CDSMP.

### Data collection

Data for the effect evaluation were collected at baseline and the three follow-up assessments by means of self-administered questionnaires and telephone interviews (see table 2). The telephone interviews were conducted independently by trained interviewers and started two weeks after the questionnaires had been sent out. Patients who announced their intention to stop during the study were asked to complete at least the last follow-up assessment (questionnaire and/or telephone interview).

Data for the process evaluation were collected from the patients by means of self-administered questionnaires. Recording forms and self-administered questionnaires were used to gather data from the CDSMP leaders (see table 3). In addition, bi-monthly structured meetings were held to allow the leaders to discuss their experiences with the various CDSMP classes.

### Power calculation

The sample size calculation was based on figures derived from Dutch research among newly diagnosed older CHF patients [21] relating to one of the primary outcome variables in the present study, general self-efficacy expectancies (GSES by Sherer and colleagues [20]). To detect a mean difference of at least 4.0 points in general self-efficacy expectancies between the intervention and control groups (SD = 11.8; equivalent to an effect size of 0.34), at least 137 patients in both the intervention and control groups were required to achieve a power of 80% at an alpha of 0.05 (one-sided). Assuming a drop-out rate of 30% during the trial, a total of 2× 180 patients have to be included in the study.

### Analysis

Descriptive techniques will be used to characterise the patients participating in the study. Data of the effect evaluation will be analysed according to the intention-to-treat and per-protocol principles. Differences between the intervention and control groups with regard to the primary and secondary outcome variables at the follow-up assessments will be analysed with multivariate techniques adjusting for possible differences in baseline scores and background characteristics. Intervention group patients will be included in the per-protocol analysis if they have attended at least four of the six programme sessions.

Data of the process evaluation will be analysed for patients and leaders by means of descriptive analysis.

## Discussion

### Progress of the study

**Figure 2:**
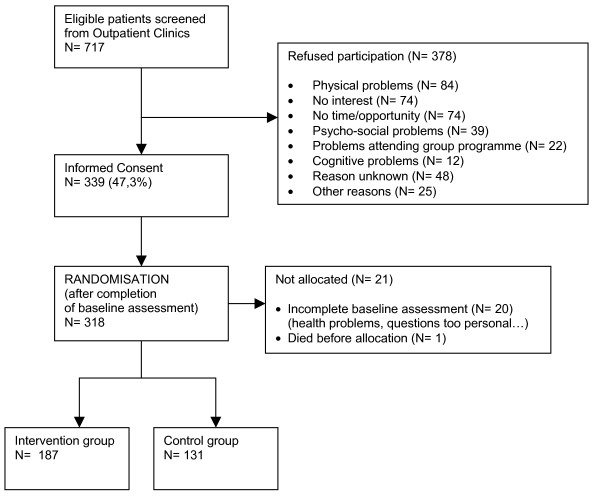
Flow of the patients until randomisation.

Between July 2004 and November 2005, a total of 717 eligible patients were selected for inclusion from six Heart Failure and/or Cardiology Outpatient Clinics, 339 of whom (47.3%) signed the informed consent form and underwent the baseline assessment (see figure 2). The remaining 378 patients refused or were unable to participate in the study for various reasons: for example, 84 patients did not participate because of physical health problems, 74 patients were not interested or felt no need to participate, and another 74 patients had no time or opportunity to participate (see figure 2). After completion of the baseline assessment, 318 patients were included in the trial; 187 patients were allocated to the intervention group and 131 to the control group.

We had calculated that the study would require a sample size of 2 × 180, including a 30% drop-out rate. A total of 187 patients were allocated to the intervention group, which was according to plan. The number of patients allocated to the control group (n = 131), however, was smaller than planned. At present, follow-up data are being collected. The exact drop-out rate and related consequences for the power calculation will become clear after completion of the data collection in January 2007.

## Competing interests

The author(s) declare that they have no competing interests.

## Authors' contributions

GK developed the project and obtained funding together with JvE and JvH. ES is the investigator and has worked together with EvH in developing the materials for the study, with input from the other authors. ES drafted the manuscript, with input from the other authors. All authors read and approved the final manuscript.

## Pre-publication history

The pre-publication history for this paper can be accessed here:



## Figures and Tables

**Table 1:** Contents of the CDSMP

**Session1**	
*Activity 1*	Introduction – Identifying common problems
*Activity 2*	Workshop overview and responsibilities
*Activity 3*	Differences between acute and chronic conditions
*Activity 4*	Introduction to cognitive symptom management
*Activity 5*	Introduction to action plans
*Activity 6*	Closing
**Session 2**	
*Activity 1*	Feedback/problem-solving session
*Activity 2*	Dealing with emotions (anger, fear, frustration)
*Activity 3*	Introduction to exercise
*Activity 4*	Making an action plan
*Activity 5*	Closing

**Session 3**	
*Activity 1*	Feedback/problem-solving session
*Activity 2*	Better breathing
*Activity 3*	Muscle relaxation
*Activity 4*	Fatigue management
*Activity 5*	Endurance exercise
*Activity 6*	Making an action plan
*Activity 7*	Closing

**Session 4 **	
*Activity 1*	Feedback/problem-solving/making an action plan
*Activity 2*	Healthy eating
*Activity 3*	Distraction
*Activity 4*	Advance directives for health care
*Activity 5*	Communication skills
*Activity 6*	Problem-solving
*Activity 7*	Closing

**Session 5**	
*Activity 1*	Feedback/problem-solving/making an action plan
*Activity 2*	Medication usage
*Activity 3*	Making informed treatment decisions
*Activity 4*	Depression management
*Activity 5*	Self-talk
*Activity 6*	Guided imagery
*Activity 7*	Closing

**Session 6**	
*Activity 1*	Feedback/problem-solving
*Activity 2*	Informing the health care team
*Activity 3*	Working with your health care professional
*Activity 4*	Looking back and planning for the future
*Activity 5*	Closing

**Table 2:** Outcome variables of the effect evaluation

**Variables**	**No. of items**	Range*	**BM**	**FU1**	**FU2**	**FU3**
**Primary outcome variables**						
General self-efficacy (GSES) [12]	16	16–80	Q	Q	Q	Q
Cardiac self-efficacy [16]						
*Control symptoms*	8	0–32	Q	Q	Q	Q
*Maintain function*	5	0–20	Q	Q	Q	Q
Perceived control [17]	7	7–35	TI	TI	TI	TI
Cognitive symptom management [20]	5	0–25	TI	TI	TI	TI
						
**Secondary outcome variables**						
Smoking behaviour	2	N/A	TI	TI	TI	TI
Drinking behaviour	2	N/A	TI	TI	TI	TI
BMI	2	N/A	TI	TI	TI	TI
Physical activity [20]	4	N/A	TI	TI	TI	TI
Self-care behaviour (EHFScBS) [21]	12	12–60	Q	Q	Q	Q
General quality of life (RAND-36) [22]						
*Physical functioning*	10	0–100	Q	Q	Q	Q
*Social functioning*	2	0–100	Q	Q	Q	Q
*Role limitations (physical problem)*	4	0–100	Q	Q	Q	Q
*Role limitations (emotional problem)*	3	0–100	Q	Q	Q	Q
*Mental health*	5	0–100	Q	Q	Q	Q
*Vitality*	4	0–100	Q	Q	Q	Q
*Pain*	2	0–100	Q	Q	Q	Q
*General health perception*	5	0–100	Q	Q	Q	Q
*Health change*	1	0–100	Q	Q	Q	Q
Cardiac quality of life (KCCQ) [23]						
*Physical limitations*	6	0–100	Q	Q	Q	Q
*Symptoms*	8	0–100	Q	Q	Q	Q
*Self-efficacy*	2	0–100	Q	Q	Q	Q
*Social interference*	4	0–100	Q	Q	Q	Q
*Quality of life*	3	0–100	Q	Q	Q	Q
Perceived autonomy	1	0–100	Q	Q	Q	Q
Symptoms of anxiety & depression [24, 25]						
*Anxiety (HADS-A)*	7	0–21	Q	Q	Q	Q
*Depression (HADS-D)*	7	0–21	Q	Q	Q	Q
Health care utilisation	7	N/A	TI	TI	TI	TI
						
**Covariates**						
Socio-demographic variables	9	N/A	TI	-	-	-
Co-morbidity [13, 28]	19	N/A	TI	-	-	-
Cognitive status (TICS) [29]	25	0–41	TI	-	-	-

BM = baseline measurement; FU1 = immediate follow-up; FU2 = follow-up after 6 months; FU3 = follow-up after 12 months; Q = questionnaire; TI = telephone interview

N/A = not applicable

* Underlined scores indicate the most favourable scores.

**Table 3:** Outcome variables of the process evaluation

**Process evaluation outcome variables**	**BDP**	**FU1**	**FU2**
**Programme performed according to protocol**			
Duration of each session	RF^l^	-	-
Deviations from the protocol	RF^l^	-	-
			
**Attendance by the patients**			
Reasons for refusal before start of the programme	TI^p^	-	-
Number of sessions attended by each patient	RF^l^	-	-
Reasons for stopping during the programme	TI^p^	-	-
			
**Adherence by patients**			
Overall adherence	RF^l^	-	-
Adherence regarding homework assignments	-	Q^p^/Q^l^	-
			
**Opinion about the programme**			
Extent of benefits from the programme	-	Q^p^	Q^p^
Strong and weak points of the programme (sessions)	RF^l^	Q^p^/Q^l^	-
General opinion about the quality of the leaders		Q^p^/Q^l^	-
General opinion about the programme	RF^l^	Q^p^/Q^l^	^-^
Recommending the programme to other patients	-	Q^p^	-
Programme contributions	-	Q^p^/Q^l^	Q^p^
Recommendations for improvement	-	Q^p^/Q^l^	-

BDP = before or during programme; FU1 = immediate follow-up; FU2 = follow-up at 12 months; RF = recording form (filled in after each session); Q = questionnaire; TI = telephone interview

Data collected from: ^l ^= leaders; ^p ^= patients

## References

[B1] Dickstein K, Jaarsma T (2005). Heart failure management programmes: delivering the message. Eur J Heart Fail.

[B2] Stewart S, MacIntyre K, Capewell S, McMurray JJ (2003). Heart failure and the aging population: an increasing burden in the 21st century?. Heart.

[B3] Clark RA, McLennan S, Dawson A, Wilkinson D, Stewart S (2004). Uncovering a hidden epidemic: a study of the current burden of heart failure in australia. Heart Lung Circ.

[B4] van Jaarsveld CH, Sanderman R, Miedema I, Ranchor AV, Kempen GI (2001). Changes in health-related quality of life in older patients with acute myocardial infarction or congestive heart failure: a prospective study. J Am Geriatr Soc.

[B5] Blue L, McMurray J (2005). How much responsibility should heart failure nurses take?. Eur J Heart Fail.

[B6] DeWalt DA, Malone RM, Bryant ME, Kosnar MC, Corr KE, Rothman RL, Sueta CA, Pignone MP (2006). A heart failure self-management program for patients of all literacy levels: a randomized, controlled trial [ISRCTN11535170]. BMC Health Serv Res.

[B7] Jaarsma T, Halfens R, Huijer Abu-Saad H, Dracup K, Gorgels T, van Ree J, Stappers J (1999). Effects of education and support on self-care and resource utilization in patients with heart failure. Eur Heart J.

[B8] Martensson J, Stromberg A, Dahlstrom U, Karlsson JE, Fridlund B (2005). Patients with heart failure in primary health care: effects of a nurse-led intervention on health-related quality of life and depression. Eur J Heart Fail.

[B9] Sethares KA, Elliott K (2004). The effect of a tailored message intervention on heart failure readmission rates, quality of life, and benefit and barrier beliefs in persons with heart failure. Heart Lung.

[B10] Schechter CB, Walker EA (2002). Improving adherence to diabetes self-management recommendations. Diabetes Spectr.

[B11] Belardinelli R, Georgiou D, Cianci G, Purcaro A (1999). Randomized, controlled trial of long-term moderate exercise training in chronic heart failure: effects on functional capacity, quality of life, and clinical outcome. Circulation.

[B12] Living with heart failure. http://www.patienthealthinternational.com/article/501796.aspx.

[B13] Health Library. http://12.31.13.50/library/healthguide/en-us/illnessconditions/topic.asp?hwid=support/tp17534.

[B14] Lorig K, Holman H, Sobel D, Laurent D, Gonzalez V, Minor M (2000). Living a healthy life with chronic conditions: self-management of heart disease, arthritis, diabetes, asthma, bronchitis, emphysema and others.

[B15] Lorig KR, Ritter P, Stewart AL, Sobel DS, Brown BW, Bandura A, Gonzalez VM, Laurent DD, Holman HR (2001). Chronic disease self-management program: 2-year health status and health care utilization outcomes. Med Care.

[B16] Lorig KR, Sobel DS, Ritter PL, Laurent D, Hobbs M (2001). Effect of a self-management program on patients with chronic disease. Eff Clin Pract.

[B17] Lorig KR, Sobel DS, Stewart AL, Brown BW, Bandura A, Ritter P, Gonzalez VM, Laurent DD, Holman HR (1999). Evidence suggesting that a chronic disease self-management program can improve health status while reducing hospitalization: a randomized trial. Med Care.

[B18] Lorig KR, Holman H (2003). Self-management education: history, definition, outcomes, and mechanisms. Ann Behav Med.

[B19] Bandura A (1997). Self-efficacy: the exercise of control.

[B20] Sherer M, Maddux JE, Mercandante B, Prentice-Dunn S, Jacobs B, Rogers RW (1982). The self-efficacy scale: construction and validation. Psychol Rep.

[B21] Kempen GI, Sanderman R, Miedema I, Meyboom-de Jong B, Ormel J (2000). Functional decline after congestive heart failure and acute myocardial infarction and the impact of psychological attributes. A prospective study. Qual Life Res.

[B22] Lorig K, Gonzalez V (1992). The integration of theory with practice: a 12-year case study. Health Educ Q.

[B23] Lorig KR, Ritter PL, Jacquez A (2005). Outcomes of border health Spanish/English chronic disease self-management programs. Diabetes Educ.

[B24] Sullivan MD, LaCroix AZ, Russo J, Katon WJ (1998). Self-efficacy and self-reported functional status in coronary heart disease: a six-month prospective study. Psychosom Med.

[B25] Arnold R (2004). Quality of life in chronic obstructive pulmonary disease and chronic heart failure: disease-specific and generic factors in adaptation to illness. PhD thesis.

[B26] Arnold R, Ranchor AV, DeJongste MJ, Koeter GH, Ten Hacken NH, Aalbers R, Sanderman R (2005). The relationship between self-efficacy and self-reported physical functioning in chronic obstructive pulmonary disease and chronic heart failure. Behav Med.

[B27] Pearlin LI, Schooler C (1978). The structure of coping. J Health Soc Behav.

[B28] Bosscher RJ, Smit JH, Kempen GI (1997). Algemene competentieverwachtingen bij ouderen: Een onderzoek naar de psychometrische kenmerken van de Algemene Competentieschaal (ALCOS) [Global expectations of self-efficacy in the elderly: An investigation of psychometric characteristics of the General Self-Efficacy Scale]. Ned Tijdschr Psychol.

[B29] Kempen GI (1992). Psychometric properties of GLAS baseline measures: a pilot study (in Dutch).

[B30] Lorig K, Stewart A, Ritter P, Gonzalez V, Laurent D, Lynch J (1996). Outcome measures for health education and other health care interventions.

[B31] Jaarsma T, Stromberg A, Martensson J, Dracup K (2003). Development and testing of the European Heart Failure Self-Care Behaviour Scale. Eur J Heart Fail.

[B32] Hays RD, Sherbourne CD, Mazel RM (1993). The RAND 36-Item Health Survey 1.0. Health Econ.

[B33] Green CP, Porter CB, Bresnahan DR, Spertus JA (2000). Development and evaluation of the Kansas City Cardiomyopathy Questionnaire: a new health status measure for heart failure. J Am Coll Cardiol.

[B34] Zigmond AS, Snaith RP (1983). The hospital anxiety and depression scale. Acta Psychiatr Scand.

[B35] Bjelland I, Dahl AA, Haug TT, Neckelmann D (2002). The validity of the Hospital Anxiety and Depression Scale. An updated literature review. J Psychosom Res.

[B36] Spinhoven P, Ormel J, Sloekers PP, Kempen GI, Speckens AE, Van Hemert AM (1997). A validation study of the Hospital Anxiety and Depression Scale (HADS) in different groups of Dutch subjects. Psychol Med.

[B37] van der Zee KI, Sanderman R (1993). Het meten van de algemene gezondheidstoestand met de RAND-36, een handleiding.

[B38] Kempen GI, Ranchor AV, Ormel J, van Sonderen E, van Jaarsveld CH, Sanderman R (2005). Perceived control and long-term changes in disability in late middle-aged and older persons: An eight-year follow-up study. Psychol Health.

[B39] Kempen GI, Meier AJ, Brouwens SF, van Deursen J, Verhey FR Telefonisch Interview Cognitieve Status (TICS): psychometrische aspecten [Psychometric properties of the Dutch version of the Telephone Interview Cognitive Status (TICS)]. Tijdschr Gerontol Geriatr.

[B40] Brandt J, Spencer M, Folstein M (1988). The Telephone Interview for Cognitive Status. NNBN.

